# Neuroprotective role of retinal SIRT3 against acute photo-stress

**DOI:** 10.1038/s41514-017-0017-8

**Published:** 2017-12-04

**Authors:** Norimitsu Ban, Yoko Ozawa, Hideto Osada, Jonathan B. Lin, Eriko Toda, Mitsuhiro Watanabe, Kenya Yuki, Shunsuke Kubota, Rajendra S. Apte, Kazuo Tsubota

**Affiliations:** 1 Laboratory of Retinal Cell Biology, Keio University School of Medicine, Shinjuku-ku, Tokyo, 160-8582 Japan; 2 Department of Ophthalmology, Keio University School of Medicine, Shinjuku-ku, Tokyo, 160-8582 Japan; 3 Department of Ophthalmology and Visual Sciences, Washington University in St. Louis School of Medicine, St. Louis, 63110 MO USA; 4 Neuroscience Program, Washington University in St. Louis School of Medicine, St. Louis, 63110 MO USA; 5 Graduate School of Media and Governance, Faculty of Environment and Information Studies, Keio University, 5322 Endo, Fujisawa, Kanagawa 252-0882 Japan; 6 Department of Developmental Biology, Washington University in St. Louis School of Medicine, St. Louis, 63110 MO USA

## Abstract

SIRT3 is a key regulator of mitochondrial reactive oxygen species as well as mitochondrial function. The retina is one of the highest energy-demanding tissues, in which the regulation of reactive oxygen species is critical to prevent retinal neurodegeneration. Although previous reports have demonstrated that SIRT3 is highly expressed in the retina and important in neuroprotection, function of SIRT3 in regulating reactive oxygen species in the retina is largely unknown. In this study, we investigated the role of retinal SIRT3 in a light-induced retinal degeneration model using SIRT3 knockout mice. We demonstrate that SIRT3 deficiency causes acute reactive oxygen species accumulation and endoplasmic reticulum stress in the retina after the light exposure, which leads to increased photoreceptor death, retinal thinning, and decreased retinal function. Using a photoreceptor-derived cell line, we revealed that reactive oxygen species were the upstream initiators of endoplasmic reticulum stress. Under SIRT3 knockdown condition, we demonstrated that decreased superoxide dismutase 2 activity led to elevated intracellular reactive oxygen species. These studies have helped to elucidate the critical role of SIRT3 in photoreceptor neuronal survival, and suggest that SIRT3 might be a therapeutic target for oxidative stress-induced retinal disorders.

## Author Summary

Sirtuins are nicotinamide adenine dinucleotide-dependent protein deacetylases. Among seven sirtuins, SIRT3 is a key regulator of mitochondrial function. However, functions of SIRT3 in the retina are largely unknown. In this study, we investigated the role of retinal SIRT3 in a mouse model of light-induced retinal degeneration, found that SIRT3 has neuroprotective role in the retina. We demonstrate that SIRT3 deficiency causes acute reactive oxygen species accumulation and endoplasmic reticulum stress in the retina after the light exposure, which leads to increased photoreceptor death, retinal thinning, and decreased retinal function. Using a photoreceptor-derived cell line, we revealed that reactive oxygen species were the upstream initiators of endoplasmic reticulum stress, and decreased superoxide dismutase 2 activity led to elevated intracellular reactive oxygen species. These results suggest that SIRT3 might be a therapeutic target for oxidative stress-induced retinal disorders.

## Introduction

Sirtuins are highly conserved nicotinamide adenine dinucleotide (NAD^+^)-dependent protein deacetylases, which modulate various metabolic responses affecting aging and longevity.^1^ We have previously reported that all seven sirtuins are highly expressed in the retina and that their expression varied with light–dark conditions.^2^

Among the seven sirtuins, SIRT3, SIRT4, and SIRT5 regulate the activity of mitochondrial enzymes and drive metabolic cycles.^3,4^ In particular, SIRT3 is considered a key regulator of mitochondrial functions.^4–6^ Initial studies revealed that SIRT3 knockout (KO) mice showed significantly increased mitochondrial protein acetylation in the liver, although there was no obvious systemic phenotype at baseline.^4^

Subsequent studies demonstrated that SIRT3 has a neuroprotective effect in murine models of age-related hearing loss^7^ and noise-induced hearing loss^8^ with reduction of local reactive oxygen species (ROS), indicating that SIRT3 might have a critical role in neurodegenerative diseases by reducing local ROS. Notably, several articles have recently demonstrated the critical role of NAD^+^ in the retina.^9,10^ Moreover, loss of function studies have demonstrated that metabolic stresses in mice that are deficient in both SIRT3 and SIRT5 induce severe photoreceptor degeneration^9^ although the molecular mechanisms are still unclear.

In this study, we investigated the molecular mechanism by which retinal SIRT3-regulated photoreceptor survival in a light-induced retinal degeneration model. We found that under conditions of acute photo-stress, SIRT3 has a neuroprotective effect in the retina. We also explored the molecular mechanisms of this effect of SIRT3 using photoreceptor-derived cells. These studies demonstrate the critical role of SIRT3 for protecting the retinal structure and function against acute photo-stress and for maintaining retinal homeostasis.

## Results

### SIRT3 was widely expressed in the retina and mainly localized to mitochondria

**Fig. 1:**
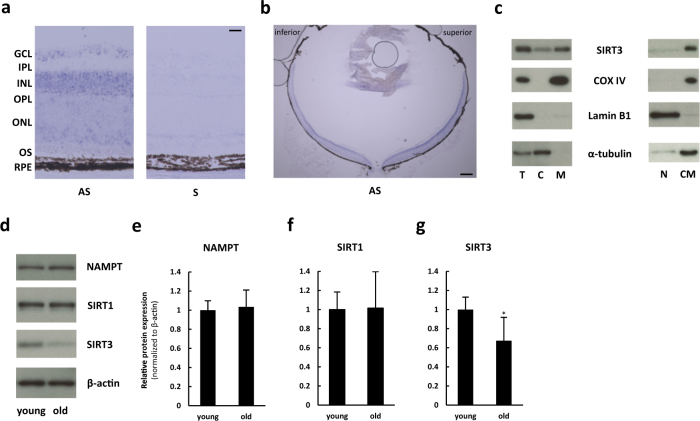
Retinal SIRT3 was mainly expressed in mitochondria, and the level was decreased with age. **a**,** b** In situ hybridization of *Sirt3* in the retina of 10-week-old C57BL6/J mice. Scale bars: **a** 20 μm **b** 200 μm. GCL ganglion cell layer, IPL inner plexiform layer, INL inner nuclear layer, OPL outer plexiform layer, ONL outer nuclear layer, OS outer segment, RPE retinal pigment epithelium, AS antisense, S sense. **c** Representative Immunoblot of SIRT3 for subcellular fraction of the retina of 10-week-old C57BL6/J mice. T total retina, C cytoplasmic, M mitochondrial, N nuclear, CM cytoplasmic and mitochondrial fractions. **d**–**g** Immunoblot of young (10 weeks old) and old (60 weeks old) C57BL6/J mice retina against nicotinamide phosphoribosyltransferase (NAMPT) **e**, SIRT1 **f**, and SIRT3 **g** (*n* = 5 for each group). *p < 0.05 by two-tailed unpaired *t*-test. Values are mean ± SD

To determine the distribution of SIRT3 in the retina, we performed *Sirt3* in situ hybridization in the retina. The results showed that *Sirt3* mRNA expression was widespread in the retina (Fig. 1a) with clear signal in the ganglion cell layer, the inner nuclear layer, and the outer nuclear layer (ONL). We did not find any qualitative differences in the expression patterns of the central versus the peripheral retina (Fig. 1b). We also assessed the subcellular localization of SIRT3 by measuring the SIRT3 protein level in each subcellular fraction. We found that the mitochondrial fraction had the highest level of SIRT3 protein expression with lower level in the cytoplasmic fraction, and non-detectable levels in the nuclear fraction (Fig. 1c).

### Aged mice showed decreased SIRT3, but not NAMPT or SIRT1, in the retina

In order to examine the effect of aging on SIRT3 expression, we examined the retinas of young (10 weeks old) and old (60 weeks old) mice. We investigated not only SIRT3 but also nicotinamide phosphoribosyltransferase (NAMPT), a key enzyme in the dominant mammalian NAD^+^ biosynthesis pathway and therefore essential for maintaining sirtuin function,^11^ and SIRT1, another sirtuin family member associated with aging^1,12^ (Fig. 1d-g). Although old mice did not show any significant reductions in NAMPT or SIRT1 in the retina (Fig. 1e,f), we found that SIRT3 protein expression was reduced in the retinas of old mice compared with those of young mice (Fig. 1g).

### SIRT3 KO retinas showed increased mitochondrial protein acetylation and mitochondrial damage

**Fig. 2:**
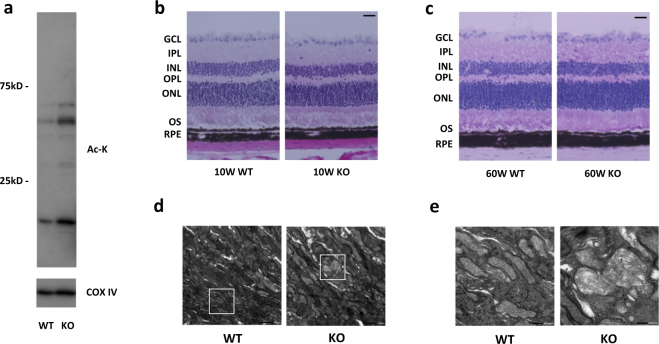
SIRT3 KO retinas showed increased acetylated protein and damaged mitochondria. **a** Representative images of Immunoblot of mitochondrial fraction isolated from 10-week-old WT and SIRT3 KO retinas against Acetylated-Lysine (Ac-K). **b**,** c** Representative HE staining images of **b** young (10 weeks old) and **c** old (60 weeks old) WT and SIRT3 KO mice retinas. Scale bars: 20 μm. GCL ganglion cell layer, IPL inner plexiform layer, INL inner nuclear layer, OPL outer plexiform layer, ONL outer nuclear layer, OS outer segment, RPE retinal pigment epithelium. **d**, **e** Electromicroscopy analysis of the morphology of mitochondria in the photoreceptor inner segments of 10-week-old WT and SIRT3 KO retinas. Mitochondria of WT retinas exhibited regularly spaced and densely packed cristae, whereas mitochondria of SIRT3 KO retinas exhibited sparse with irregularly arranged cristae. Scale bars: **d** 1 μm **e** 200 nm

In order to examine the functional importance of SIRT3 in the retina, we analyzed the retinal phenotypes of SIRT3 KO mice. First, we confirmed the deletion of SIRT3 in the retina of SIRT3 KO mice by real-time PCR and immunoblot analyses (Supplementary Fig. 1a,b). We then demonstrated increased acetylation of mitochondrial proteins isolated from SIRT3 KO retinas (Fig. 2a), indicating that SIRT3 acts as a deacetylase in the retina as it does in other tissues and organs.^4–6^

Next, we analyzed the retinal histology of 10- and 60-week-old SIRT3 KO mice by light microscopy but did not detect any obvious changes compared with wild-type (WT) mice at either age (Fig. 2b,c). Electroretinography (ERG) of 10- and 60-week-old SIRT3 KO mice also showed no significant change in retinal function compared with WT mice (data not shown). However, electron microscopy (EM) revealed that the mitochondria in the photoreceptor inner segments of SIRT3 KO retinas were dysmorphic and exhibited sparse, irregularly arranged cristae, whereas mitochondria of WT retinas exhibited regularly spaced and densely packed cristae (Fig. 2d,e).

### SIRT3 KO retinas showed increased cell death and neuroretinal degeneration after the light exposure

**Fig. 3:**
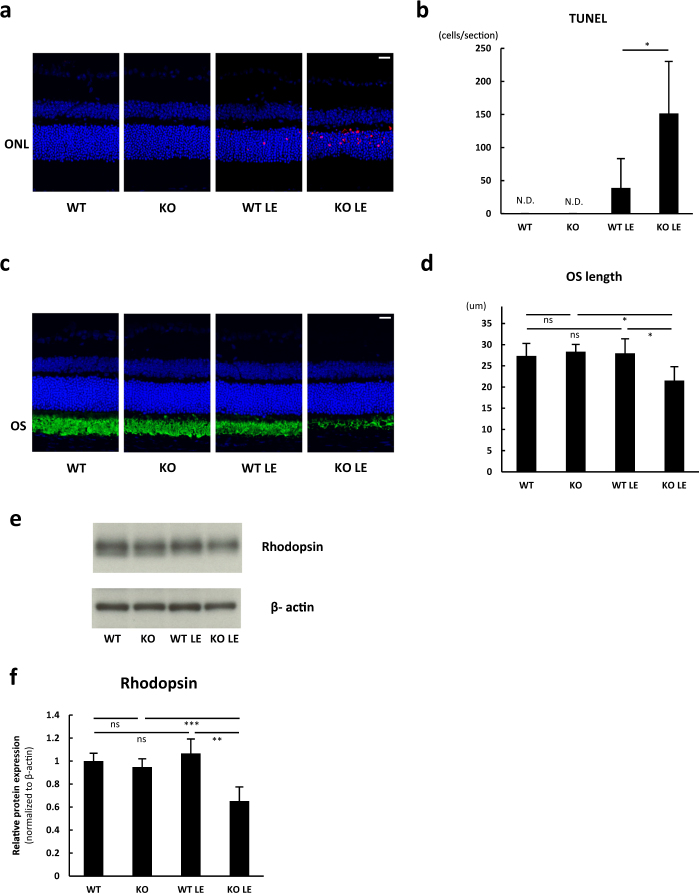
SIRT3 KO retinas showed increased cell death after the light exposure (LE). **a** Representative images of terminal deoxynucleotidyl transferase dUTP nick end labeling (TUNEL) stain for WT and SIRT3 KO retinas without and with LE (36 h after LE). Blue: DAPI, Red: TUNEL. Scale bars: 20 μm. ONL outer nuclear layer. **b** Comparison of TUNEL-positive cell counts per section (*n* = 4 per group). *p < 0.05 by two-tailed unpaired *t*-test. **c** Representative images of Immunostaining of Rhodopsin for WT and SIRT3 KO retinas without and with LE (36 h after LE). Blue: DAPI, Green Rhodopsin. Scale bars: 20 μm. OS outer segment. **d** Comparison of outer segment (OS) length (36hr after LE, WT: *n* = 4, KO: *n* = 4, WT LE: *n* = 5, KO LE: *n* = 6). *p < 0.05 by one-way ANOVA with post hoc Tukey’s HSD test. **e, f** Immunoblot of Rhodopsin for WT and SIRT3 KO retinas without and with LE (36 h after LE, WT: *n* = 4, KO: *n* = 4, WT LE: *n* = 5, KO LE: *n* = 6). **p < 0.01 and ***p < 0.001 by one-way ANOVA with post hoc Tukey’s HSD test. Values are mean ± SD

To investigate the SIRT3 function in the retina under the metabolic stress, we analyzed the susceptibility of WT and SIRT3 KO retinas to the light exposure. In this model, photo-toxicity rapidly induces ROS, which causes subsequent photoreceptor cell death.^13,14^ We found a significant increase in terminal deoxynucleotidyl transferase dUTP nick end labeling (TUNEL)-positive apoptotic cells in SIRT3 KO retinas compared with WT retinas 36 h after the light exposure (Fig. 3a,b). Furthermore, we found that light-exposed SIRT3 KO retinas showed significant shortening of outer segments (OS), where the photopigments such as rhodopsin are concentrated (Fig. 3c,d). Consistently, we also observed a significant reduction of rhodopsin protein 36 h after the light exposure, indicating that SIRT3 KO mice exhibited more severe photoreceptor degeneration in response to acute photo-stress (Fig. 3e,f). Based on these results, we concluded that SIRT3 KO retinas were more vulnerable to the light-induced stress.

### SIRT3 KO retinas showed decreased retinal function and ONL thickness after the light exposure

**Fig. 4:**
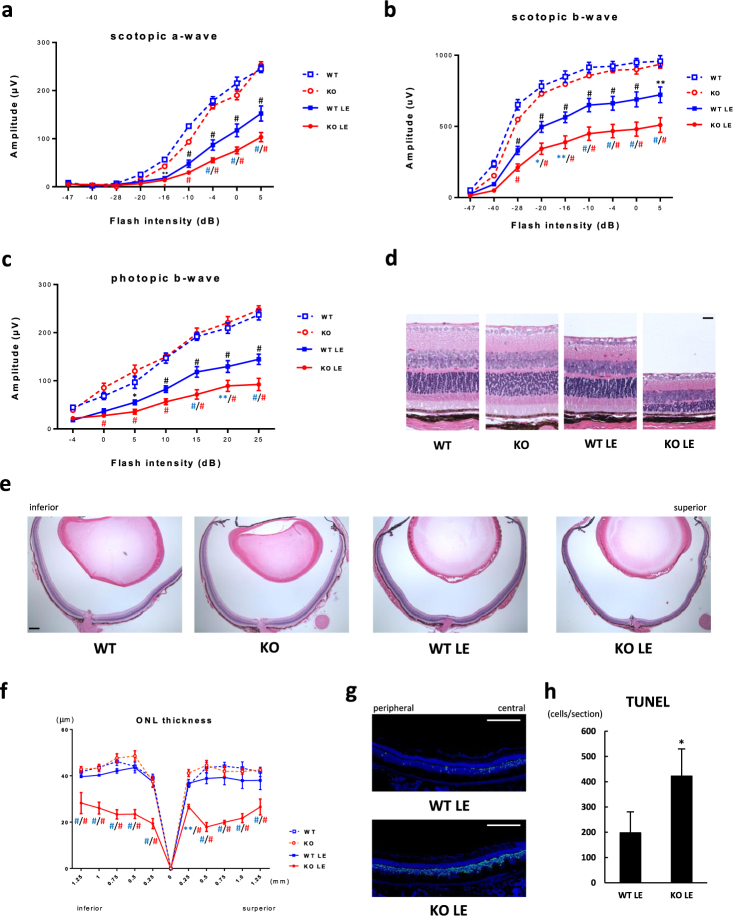
SIRT3 KO retinas showed decreased retinal function and outer nuclear layer (ONL) thinning after the light exposure (LE). **a**–**c** Electroretinogram (ERG) of WT and SIRT3 KO retinas with 129 background without and with LE (4 days after LE; WT (blue square with dashed line): *n* = 4, KO (red circle with dashed line): *n* = 4, WT LE (blue square with solid line): *n* = 8, KO LE (red circle with solid line): *n* = 10) **a** Scotopic a-wave amplitude of WT and SIRT3 KO retinas with 129 background without and with LE. **b** Scotopic b-wave amplitude of WT and SIRT3 KO retinas with 129 background without and with LE. **c** Photonic b-wave amplitude of WT and SIRT3 KO retinas with 129 background without and with LE. *p < 0.05, **p < 0.01, and #*p* < 0.001 by two-way ANOVA with post hoc Tukey’s HSD test (black: WT vs WT LE, blue: WT LE vs KO LE, red: KO vs KO LE). **d**,** e** Representative HE staining images of WT and SIRT3 KO retinas with 129 background without and with LE (7 days after LE). Scale bars: **d** 20 μm **e** 200 μm. **f** Outer nuclear layer (ONL) thickness of WT and SIRT3 KO retinas with 129 background without and with LE (7 days after LE; WT (blue square with dashed line): *n* = 4, KO (red circle with dashed line): *n* = 4, WT LE (blue square with solid line): *n* = 4, KO LE (red circle with solid line): *n* = 5). *p < 0.05, **p < 0.01, and #*p* < 0.001 by two-way ANOVA with post hoc Tukey’s HSD test (blue: WT LE vs KO LE, red: KO vs KO LE). **g** Representative images of terminal deoxynucleotidyl transferase dUTP nick end labeling (TUNEL) stain for WT and SIRT3 KO retinas with 129 background 36 h after LE. Blue: DAPI, Green: TUNEL. Scale bars: 250 μm. **h** Comparison of TUNEL-positive cell counts per section (*n* = 6 for WT LE and *n* = 4 for KO LE). *p < 0.05 by two-tailed unpaired *t*-test. Values are mean ± SE for ERG analysis, and mean ± SD for ONL thickness and TUNEL analysis, respectively

Although SIRT3 KO retinas appeared to be more vulnerable to damage following the light exposure, we did not observe any clear differences in retinal function measured by ERG with the C57BL6/J (B6) background. We hypothesized that genetic background of SIRT3 KO mice was responsible for the subtle phenotype of light-induced retinal degeneration. It has been previously demonstrated that C57BL/6 J mouse strain is resistant to the light-induced retinal degeneration owing to a mutation in the RPE65 that leads to a substitution of leu450met.^15^ This substitution causes a significant slowing of the RPE-driven visual cycle in these mice that makes them resistant to light-induced retinal degeneration.^15^ In order to circumvent this effect, we further analyzed SIRT3 KO mice with the 129S6/SvEvTac (129) genetic background, a strain that is more susceptible to light-induced degeneration.^15^ First, we confirmed deletion of SIRT3 in the retinas of these SIRT3 KO mice with 129 background by real-time PCR and immunoblot analyses (Supplementary Fig. 2a,b). SIRT3 KO retinas with 129 background had a significant decrease in the amplitudes of their scotopic a-waves (Fig. 4a), scotopic b-waves (Fig. 4b), and photopic b-waves (Fig. 4c) by ERG 4 days after the light exposure compared with WT retinas with the same 129 background. Consistently, we also found that SIRT3 KO retinas with 129 background had significant thinning of the ONL by histology 7 days after the light exposure compared with WT retinas with the same 129 background (Fig. 4d–f). We further confirmed that the more severe thinning of ONL in SIRT3 KO retinas was owing to increased apoptotic cells in the ONL compared with WT retinas 36 h after the light exposure (Fig. 4g,h).

### SIRT3 KO retinas showed increased ROS and ER stress after the light exposure

**Fig. 5:**
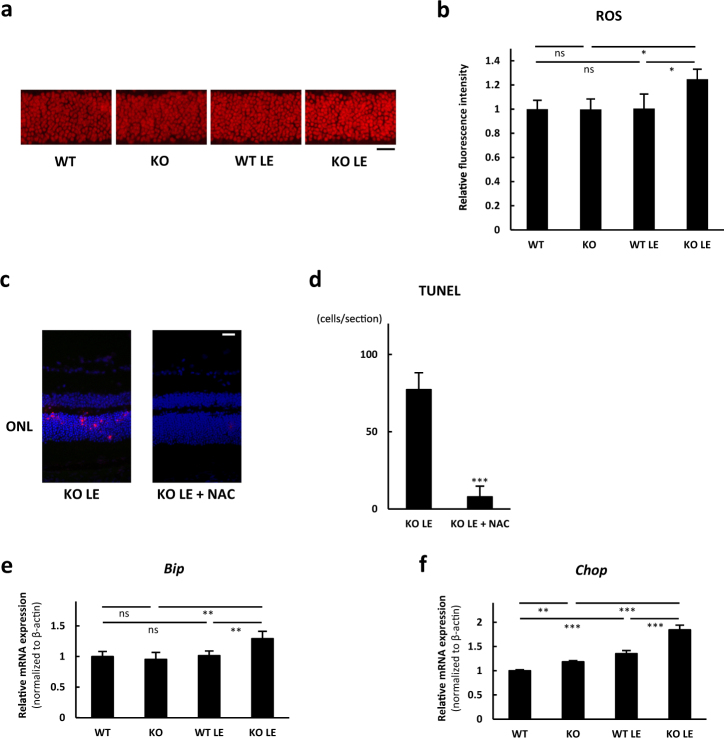
SIRT3 KO retinas showed increased reactive oxygen species (ROS) and endoplasmic reticulum (ER) stress after the light exposure (LE). **a**,** b** Comparison of in vivo DHE staining for WT and SIRT3 KO outer nuclear layers (ONL) without and with LE (1 h after LE; WT *n* = 4, KO *n* = 4, WT LE *n* = 4, KO LE *n* = 5). Scale bars: 20 μm. *p < 0.05, by one-way ANOVA with post hoc Tukey’s HSD test. **c** Representative images of terminal deoxynucleotidyl transferase dUTP nick end labeling (TUNEL) stain for SIRT3 KO retinas 36 h after the light exposure with control vehicle or N-Acetyl-l-cysteine (NAC) treatment. Blue: DAPI, Red: TUNEL. Scale bars: 20 μm. ONL outer nuclear layer. **d** Comparison of TUNEL-positive cell counts per section (*n* = 4 for control group and *n* = 6 for NAC treatment group). ***p < 0.001 by two-tailed unpaired *t*-test. **e**, **f** Expression of ER stress markers in WT and SIRT3 KO retinas without and with LE. Relative mRNA expression of **e** *Bip* and **f** *Chop* in the retina (1 h after LE; WT: *n* = 4, KO: *n* = 4, WT LE: *n* = 4, KO LE: *n* = 5). **p < 0.01 and ***p < 0.001 by one-way ANOVA with post hoc Tukey’s HSD test. Values are mean ± SD

To elucidate the mechanisms underlying the vulnerability of SIRT3-deficient retina to light stress, we analyzed both light-induced ROS levels^16^ and endoplasmic reticulum (ER) stress in the retina because not only ROS but also ER stress are considered to be critical factors for neurodegenerative diseases.^16,17^ The result showed that ROS were significantly increased in SIRT3 KO retinas 1 h after the light exposure compared with WT retinas (Fig. 5a,b). To elucidate the contribution of ROS in light-induced retinal degeneration, we administered an antioxidant, N-Acetyl-l-cysteine (NAC), before the light exposure. NAC treatment was associated with a significant decrease in TUNEL-positive cells in SIRT3 KO retinas 36 h after the light exposure (Fig. 5c,d). We also found that ER stress markers such as Binding immunoglobulin protein (*Bip*) and C/EBP homologous protein (*Chop*) were significantly increased in SIRT3 KO retinas 1 h after the light exposure compared with WT retinas (Fig. 5e,f). These results suggest that both ROS and ER stress may be involved in the vulnerability of the SIRT3 KO retinas to acute photo-stress.

### ROS is upstream of and leads to increased ER stress in photoreceptor cells

**Fig. 6:**
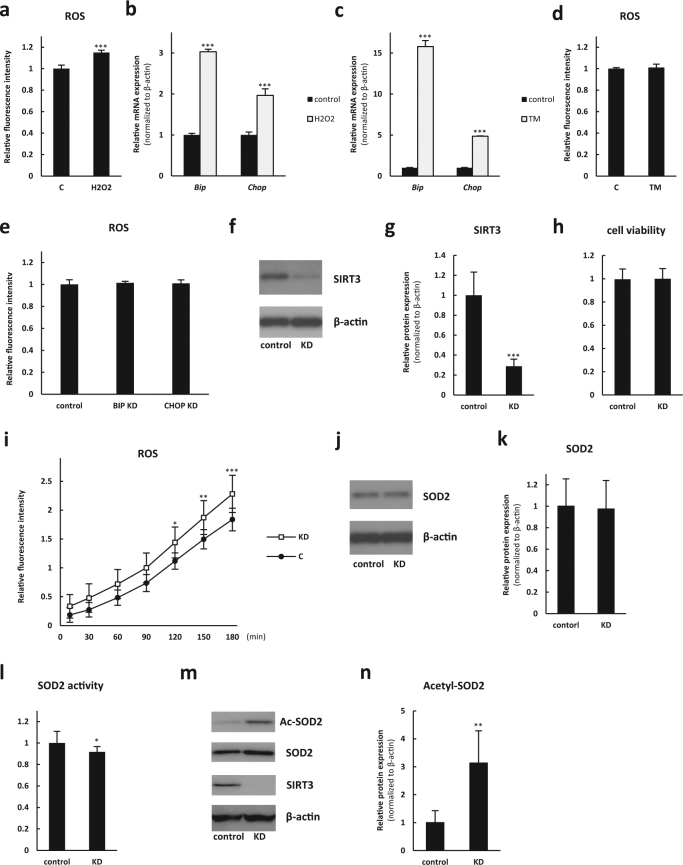
SIRT3 knockdown 661 W cells showed elevated reactive oxygen species (ROS) levels and decreased superoxide dismutase 2 (SOD2) activity. **a** in vitro ROS measurement 2 h after H_2_O_2_ treatment (*n* = 5 for each group). **b** Relative mRNA expression of *Bip* and *Chop* 2 h after H_2_O_2_ treatment (*n* = 5 for each group). **c** Relative mRNA expression of *Bip* and *Chop* 3 h after tunicamysin (TM) treatment (*n* = 5 for each group). **d** in vitro ROS measurement 3 h after TM treatment. (*n* = 5 for each group). ***p < 0.001 by two-tailed unpaired *t*-test. **e** in vitro ROS measurement after transfection with control siRNA, *Bip* siRNA (BIP KD), and *Chop* siRNA (CHOP KD) (*n* = 6 for each group). No significant difference by one-way ANOVA with post hoc Tukey’s HSD test. **f**,** g** Immunoblot against SIRT3 of 661 W cells with negative control shRNA (control) and SIRT3 knockdown (KD) (*n* = 6 for each group). ***p < 0.001 by two-tailed unpaired *t*-test. **h** Cell viability assay of 661 W cells transfected with control and SIRT3 KD (*n* = 5 for each group). **i** ROS assay of control and SIRT3 KD 661 W cells (*n* = 10 for each group). *p < 0.05, **p < 0.01, and ***p < 0.001 by two-way ANOVA with post hoc Bonferroni’s multiple comparisons test. **j**,** k** Immunoblot against SOD2 of control and SIRT3 KD 661 W cells (*n* = 5 for each group). **l** SOD2 activity assay of control and SIRT3 KD 661 W cells (*n* = 12 for each group). *p < 0.05 by two-tailed unpaired *t*-test. **m**,** n** Immunoblot against acetylated SOD2 (Ac-SOD2) of control and SIRT3 KD 661 W cells (*n* = 5 for each group). **p < 0.01 by two-tailed unpaired *t*-test. Values are mean ± SD

We observed a significant increase in both ROS and ER stress in SIRT3 KO retinas after the light exposure. In order to understand the relationship between ROS and ER stress, we used the 661 W photoreceptor-derived cell line. We administered H_2_O_2_ to raise ROS levels (Fig. 6a) and observed concomitant induction of the ER stress markers such as *Bip* and *Chop* by real-time PCR (Fig. 6b). In contrast, tunicamycin (TM), which is known to induce ER stress (Fig. 6c), did not induce ROS (Fig. 6d). Lack of ROS level changes with either Bip or Chop deficiency were also confirmed by knocking down these molecules in the 661 W cells (Fig. 6e and Supplementary Fig. 3a,b). These results suggest that ROS accumulation is upstream of and induces ER stress.

### SIRT3 knockdown in 661W cells led to increased ROS and decreased SOD2 activity

To elucidate the cause of enhanced generation of ROS in SIRT3 KO retinas, we knocked down SIRT3 in the 661 W photoreceptor-derived cell line with shRNA. Reduced SIRT3 expression in the shRNA-transfected cells was confirmed by immunoblotting (Fig. 6f,g). Although SIRT3 knockdown did not affect cell viability (Fig. 6h), it caused a significant increase in ROS generation (Fig. 6i).

To investigate the mechanism that caused increased ROS in SIRT3 knockdown cells, we analyzed superoxide dismutase 2 (SOD2), which is a known SIRT3 deacetylation target^18,19^ as well as major regulator of ROS in the mitochondria.^20^ Although the protein level of SOD2 was not significantly different between control and SIRT3 knockdown cells (Fig. 6j,k), SOD2 activity was deceased in SIRT3 knockdown cells compared with control (Fig. 6l). Moreover, acetylated SOD2 level was significantly increased in SIRT3 knockdown cells (Fig. 6m,n).

## Discussion

In this study, we have demonstrated that SIRT3 acts as a deacetylase in the retina, and that SIRT3 deficiency induces mitochondrial change in the retina. We also demonstrated that SIRT3 KO retinas were more susceptible to acute photo-stress, exhibiting accelerated photoreceptor death and degeneration with increased ROS accumulation and ER stress in the retina. Moreover, our in vitro experiments showed that elevated ROS level caused by SIRT3 deficiency was at least partially due to decreased SOD2 activity.

We found that old mice showed decreased SIRT3 protein levels in the retina, but no significant difference in NAMPT or SIRT1 protein levels. This was consistent with previous reports showing that elderly individuals exhibited lower SIRT3 protein levels in skeletal muscle than young subjects.^12^ SIRT3 deficiency decreases mitochondrial function with age, which is related to tumor-permissive phenotype.^21^ Other age-related diseases such as diabetes and cardiac diseases may also be related to the SIRT3 function.^22–24^ Based on these results, we hypothesized that reduced SIRT3 expression might cause structural and functional changes in the aged retina. To validate this hypothesis, we analyzed the retinas of aged SIRT3 KO mice and found no obvious phenotype in aged SIRT3 KO retinas at baseline, suggesting a redundancy or overlap in the functions of mitochondrial sirtuins under baseline homeostatic conditions.

In contrast to baseline conditions, we demonstrated that SIRT3 KO retinas were highly susceptible to acute photo-stress. SIRT3 KO retinas exhibited accelerated photoreceptor death and degeneration, suggesting that mitochondrial SIRT3 function was critical to photoreceptor survival in the context of acute metabolic stress. The administration of NAC, an antioxidant, attenuated the acute cytotoxicity under SIRT3-deficient conditions, creating the possibility that the effects of SIRT3 deficiency may be partially mitigated by exogenous antioxidants. Moreover, we focused on ER stress as well as ROS in our light-induced retinal degeneration model because ER stress could mediate cell death via activation of the CHOP-dependent apoptotic pathway.^25^ In retinal diseases including retinitis pigmentosa, ER stress has been implicated in photoreceptor cell death.^26^ In our model, we found that SIRT3 KO retinas showed increased ER stress as well as increased ROS after the light exposure.

To elucidate the molecular mechanisms by which SIRT3 deficiency caused retinal neurodegeneration, we investigated the causal relationship between ROS and ER stress. ROS induction in vitro resulted in increased ER stress while the changes were clear but of a smaller magnitude in vivo, although the ROS production induced by H_2_O_2_ in vitro and by light exposure in vivo were almost the same level. This may be because of the usage of dihydroethidium (DHE) and CM-H2DCFDA for ROS detection; light-induced retinal ROS in vivo were measured by DHE that detects superoxide, whereas ROS of 661 W cells in vitro were measured by CM-H2DCFDA that detects hydrogen peroxide, hydroxyl radical, peroxyl radical, and peroxynitrite anion. Because ROS are also involved in pathogenesis of various eye diseases such as diabetic retinopathy,^27^ glaucoma,^28,29^ and age-related macular degeneration,^30^ it would be interesting to study the influence of SIRT3 deficiency in these models in the future.

In addition, in vitro results also showed that elevated ROS level was partially due to decreased SOD2 activity. A previous study demonstrated that SOD2 was one of the deacetylation targets of SIRT3, and its function was activated by deacetylation.^18,19^ Our results showed the reduction of SOD2 activity in SIRT3 knock down cells with significant increase of acetylated SOD2, indicating that SOD2 activity was modulated by deacetylation.

In conclusion, we demonstrated that SIRT3 deficiency caused acute ROS increase in the retina after the light-induced stress that led to increased photoreceptor death, retinal thinning, and decreased retinal function. Our results suggest that SIRT3 contributes to retinal neuroprotection by regulating ROS through SOD2, which in turn regulates ER stress. Our findings help clarify the important role of SIRT3 in retinal neurodegeneration and offer the possibility of modulating SIRT3 activity as a novel therapeutic avenue for oxidative stress-induced retinal diseases such as age-related macular degeneration.

## Materials and methods

### Animal

All animal experiments were conducted in accordance with ARVO (Association for Research in Vision and Ophthalmology) Statement for the Use of Animals in Ophthalmic and Vision Research after approval by the Animal Studies Committee of Keio University School of Medicine or Washington University in St. Louis School of Medicine. The mice were kept in an air-conditioned room (22 ± 1 °C) under a 12-h light/12-h dark cycle. C57BL6/J (B6) mice were purchased (CLEA Japan, Tokyo, Japan) and used at 10 or 60 weeks of age. SIRT3 KO mice, which had been backcrossed for more than 10 generations with C57BL6/J (B6) mice, were kindly given by Dr. Johan Auwerx (Ecole Polytechnique Fédérale Lausanne, Switzerland). We used 8- to 12-week-old SIRT3 KO mice with B6 background for light-induced retinal degeneration experiments. SIRT3 KO mice with 129S6/SvEvTac (129) background were purchased (The Jackson Laboratory, Bar Harbor, MA, USA) and used for light-induced retinal degeneration experiments at 8–12 weeks of age. We did not use any statistical methods to estimate sample size. No randomization was done in this study. The investigator was blinded to the group allocation during the experiment.

### Light-induced retinal degeneration model

The Light-induced retinal degeneration experiments were performed as described previously.^31^ In brief, before light exposure, mice were dark-adapted by maintaining them in complete darkness for 12 h. Then, pupils were dilated with a mixture of 0.5% tropicamide and 0.5% phenylephrine (Mydrin-P; Santen Pharmaceutical, Osaka, Japan) just before exposure to the light. We used 10,000 lux (WT and SIRT3 KO mice with B6 background) or 13,000 lux (WT and SIRT3 KO mice with 129 background) from a white fluorescence lamp for 3 h (WT and SIRT3 KO mice with B6 background) or 4 h (WT and SIRT3 KO mice with 129 background) in a dedicated exposure box. After the light exposure, the mice were returned to their cages and maintained under normal light cycle until they were euthanized at the time of sampling at various time points, according to each experimental protocol. For the experiments with an antioxidant, NAC (Nacalai Tesque, Kyoto, Japan), mice were separated into two groups and intraperitoneally injected with either NAC diluted with phosphate-buffered saline (PBS) (500 mg/kg) or vehicle, twice, before dark adaptation and before light exposure.^14^

### In situ hybridization

Eyes were enucleated and embedded in OCT compound (Sakura Finetek, Tokyo, Japan) and immediately flash frozen on dry ice. Cryosections (10 μm) were fixed with 4% paraformaldehyde in PBS for 10 min at room temperature. After the fixation, sections were rinsed 10 min in PBS. Prior to in situ hybridization, the sections were treated with 10 mg/ml proteinase K (Roche Applied Science, Penzberg, Germany) at 37 °C for 7 min and then hybridized with sense or antisense DIG-labeled RNA probes at 65 °C overnight. The hybridization signals were detected using alkaline phosphatase-conjugated anti-DIG antibody (Roche Applied Science) and NBT/BCIP (Roche Applied Science) as the chromogen. Sense and antisense digoxigenin-labeled RNA probes were generated using in vitro transcription with a DIG RNA labeling kit (Roche Applied Science). The probe templates were generated by RT-PCR using the following primers. The amplified fragments were cloned into modified BluescriptSK(+).

### Subcellular fractionation of the retina

To separate the mitochondrial and cytoplasmic fractions of retina lysates, Mitochondria/Cytosol Fractionation kit (Bio Vision, San Francisco, CA, USA) was used according to the manufacturer’s protocol. To separate nuclear fraction and others, NE-PER Nuclear and Cytoplasmic Extraction Reagents (Thermo Fisher Scientific, Waltham, MA, USA) were used according to the manufacturer’s protocol. For immunoblot analysis, 5 μg of protein from each subcellular fraction and total retinal lysate were used.

### Immunoblot analyses

Samples of tissue or cell extract were separated by SDS–polyacrylamide gel electrophoresis, and the proteins were transferred to a polyvinylidene fluoride membrane (Immobilon-P; Millipore, Bedford, MA, USA) in a Trans-Blot SD Cell (BIO-RAD, Hercules, CA, USA). The membrane was blocked with 0.5% TSA Blocking Reagent (PerkinElmer Life Sciences, Waltham, MA, USA), incubated overnight at 4 °C with one of the primary antibodies—anti-NAMPT (1:1000,#A300-372A; Bethyl Laboratories Inc, Montgomery, TX, USA), anti-SIRT1 (# 07-131; Millipore), anti-SIRT3 (#5490; Cell Signaling Technology), anti- β-actin (#4970; Cell Signaling Technology), anti-Cox IV (#ab16056;Abcam, Cambridge, UK), anti- LaminB1 (#ab16048; Abcam), anti-α-tublin (#T9026; Sigma-Aldrich, St. Louis, MO, USA), anti-acetyllysine (#9441; Cell Signaling Technology), anti-SOD2 (#ADI-SOD-111; Enzo life Sciences, NY, USA), and anti-acetylated SOD2^19^ (#ab214675; Abcam)—and then incubated with horseradish peroxidase-conjugated secondary antibody (Jackson Immuno Research Laboratories Inc., West Grove, PA, USA) for 1 h. The signals were detected using ECL Western Blotting Detection Reagents (GE healthcare Limited, Buckinghamshire, UK). Signal intensities were quantified using the ImageJ program and normalized to β-actin.

### RNA isolation and real-time PCR

The samples were placed in TRIzol reagent (Life Technologies, Carlsbad, CA, USA) to extract total RNA. One μg of the total RNA was added to the Super Script VILO cDNA Synthesis Kit (Life Technologies) and reverse-transcribed according to the manufacturer’s protocol. PCR was performed using the StepOnePlus Real-Time PCR system (Life Technologies), and the mRNA was quantified using the delta CT method. Each mRNA level was normalized to β-actin as an internal control. The primers used in this study are *Sirt3* 5′-TACAGGCCCAATGTCACTCA-3′ and 5′-CTTCGACAGACCGTGCATGTA-3′, β-actin 5′-AGGTCATCACTATTGGCAACGA-3′ and 5′-GTTTCATGGATGCCACAGGA-3′, *Bip* 5′-TGCAGCAGGACATCAAGTTC-3′ and 3′-TTTCTTCTGGGGCAAATGTC-5′, *Chop* 5′-CTGGAAGCCTGGTATGAGGA-3′ and 3′-GGACGCAGGGTCAAGAGTAG-5′.

### Immunohistochemistry

The cryosections (10 μm) described above were fixed with 4% paraformaldehyde in PBS for 10 min at room temperature. After fixation, sections were stained with hematoxylin and eosin, or incubated overnight at 4 °C mouse anti-rhodopsin antibody (Thermo Fisher Scientific) followed by Alexa 488-conjugated goat anti-mouse or rabbit IgG (Invitrogen, Carlsbad, CA, USA) at room temperature for 1 h. Nuclei were counterstained with DAPI (Sigma-Aldrich). All sections were examined under a confocal microscope equipped with a digital camera (Olympus, Tokyo, Japan). OS lengths were measured in the inferior retina at 500 µm from optic nerve using Image J.

### Transmission electron microscopy examination

Eye sample were immediately fixed with 2.5% glutaraldehyde in 100 mM PBS (pH 7.4), immersed for 4 h at 4 °C, and then washed three times with 0.1 M PBS solution. The samples were then post-fixed in 2% osmium tetroxide, dehydrated in a series of ethanol and propylene oxide, and embedded in epoxy resin. Sections were stained with uranyl acetate and lead citrate, examined, and photographed using an electron microscope (model 1200 EXII; JEOL, Tokyo, Japan).

### ERG

ERG was performed as previously described^9,32^ using the UTAS-E3000 Visual Electrodiagnostic System running EM for Windows (LKC Technologies, Gaithersburg, MD, USA). In brief, we extracted quantitative measurements from the ERG wave forms using an existing Microsoft Excel macro that defines the a-wave amplitude as the difference between the average pre-trial baseline and the trough of the average trace and defines the b-wave amplitude as the difference between the trough to the peak, without subtracting oscillatory potentials.

### TUNEL staining

TUNEL staining was performed according to the manufacturer’s protocol (Apoptag Fluorescein in situ Apoptosis Detection Kit; Chemicon, Temecula, CA, USA) using cryosections (10 μm) described above. Fluorescein images were obtained using an Axio Imager (Carl Zeiss, Oberkochen, Germany), and TUNEL-positive cells were counted in the retina of the sections.

### In vivo ROS measurement

The protocol for measuring ROS was described previously.^29,33^ In brief, unfixed cryosections (10 μm) described above were incubated with 5 μM DHE (Invitrogen-Molecular Probes, Eugene, OR, USA) for 20 min at 37 °C. Sections were examined using an Axio Imager (Carl Zeiss) with Filter set 43 HE (Excitation; BP550/25HE, Emission; BP605/70HE, Carl Zeiss). The intensity of staining in the ONL was measured using Image J. All procedures for each sample, from preparing the animals to taking the photographs, were performed at the same time in parallel.

### Cell culture

The mouse cone-derived cell line 661 W cells (generously provided by Dr Miayyad Al-Ubaidi, University of Oklahoma, OK, USA) were maintained in low-glucose Dulbecco’s modified Eagle’s medium (#08456-65; Nacalai tesque, Kyoto, Japan) supplemented with 10% fetal bovine serum (Life technologies, Carlsbad, CA, USA), 100 unit/ml penicillin and 100 μg/ml streptomycin (Sigma-Aldrich, St. Louis, MO, USA) at 37 °C under a humidified atmosphere of 5% CO2.

### In vitro ROS induction by H_2_O_2_

To induce ROS to 661 W cells, we added H_2_O_2_ (Thermo Fisher Scientific) to the cell culture medium at a final concentration of 125 nM. All experiments were performed 2 h after the treatment.

### In vitro ER stress induction by TM

To induce ER stress in 661 W cells, we added TM (#T7765;Sigma-Aldrich) at final concentration of 1 μg/ml. All experiments were performed 3 h after the treatment.

### In vitro ROS measurement

The protocol for measuring ROS was described previously.^34^ In brief, intracellular ROS levels were determined using the fluorescent dye, CM-H2DCFDA (Invitrogen). A 2 mM stock solution of CM-H2DCFDA was reconstituted in anhydrous dimethyl sulfoxide (DMSO) (Sigma-Aldrich), and added to the culture at 10 μM. After incubation at 37 °C for the appropriate amount of time, fluorescence was measured (excitation wavelength, 485 nm; emission wavelength, 528 nm) every 30 min after starting the incubation using Spectra Max Paradigm (Molecular Devices LLC, Sunnyvale, CA, USA). Time 0 corresponded to the timepoint of adding CM-H2DCFDA to the sample. We used the measurement values at the timepoint of 90 min when we use the representative measurements.

### In vitro knock down

To knock down BIP or CHOP in 661 W cells, we introduced either control siRNA (Negative Control Lo GC, Thermo Fisher Scientific), or *Bip* siRNA (Hspa5 MSS204938, Thermo Fisher Scientific) or *Chop* siRNA (Ddit3 MSS273951, Thermo Fisher Scientific), using Lipofectamine RNAiMAX Reagent (Thermo Fisher Scientific), according to the manufacturer’s protocol, and incubated for 24 h before collecting the cells for experiments. To knock down SIRT3 in 661 W cells, we used *Sirt3* shRNA (#NM_022433; Qiagen, Hilden, Germany) with Attractene Transfection Reagent (Qiagen) according to the manufacturer’s protocol. In brief, 661 W cell suspension and transfection complexes were mixed and seeded to 24-well plate, and then transfected cells are incubated for 48 h before the experiments.

### Cell viability assay

To determine cell survival, we used MTT assay (#M5655, Sigma-Aldrich) according to manufacturer’s instructions. In brief, we added 5 mg/ml MTT stock solution to each culture plate at a working concentration of 500 μg/ml. After incubating 3 h at 37 °C, we removed medium, added DMSO, and then measured absorbance at 570 nm using the Spectra Max Paradigm (Molecular Devices LLC).

### In vitro SOD2 activity assay

SOD2 activity of the retina was measured using the SOD assay kit (Dojindo, Kumamoto, Japan) according to manufacturer’s instructions. In brief, to directly measure SOD2 activity, SOD1 was inhibited by the addition of 1 mM KCN and preincubated at room temperature for 5 min. The samples were measured colorimetrically at 440 nm using a microplate reader, Spectra Max Paradigm (Molecular Devices LLC).

### Statistics

All statistical tests were performed using IBM SPSS statistics Ver.19 (IBM, Armonk, NY, USA). Prior to all data analysis, we assessed the normality of the data graphically and with the Kolmogorov–Smirnov test. All the results are presented as the mean ± SD except for ERG data that was presented as the mean ± SE. *P* value < 0.05 was considered statistically significant.

### Data availability statement

The data that support the findings of this study are available from the corresponding author upon reasonable request.

## Electronic supplementary material

Supplementary Figure

## Electronic supplementary material

**Supplementary information** accompanies the paper on the *npj Aging and Mechanisms of Disease* website (10.1038/s41514-017-0017-8).
