# Supplementary material for: Neuroprotective role of retinal SIRT3 against acute photo-stress
Source: NPJ Aging Mech Dis. 2017 Dec 4;3:19. doi: 10.1038/s41514-017-0017-8 (PMC5712523; doi:10.1038/s41514-017-0017-8)
Supplement: Supplementary file 1 — Supplementary Figure [file 41514_2017_17_MOESM1_ESM.pptx]

## Slide 1
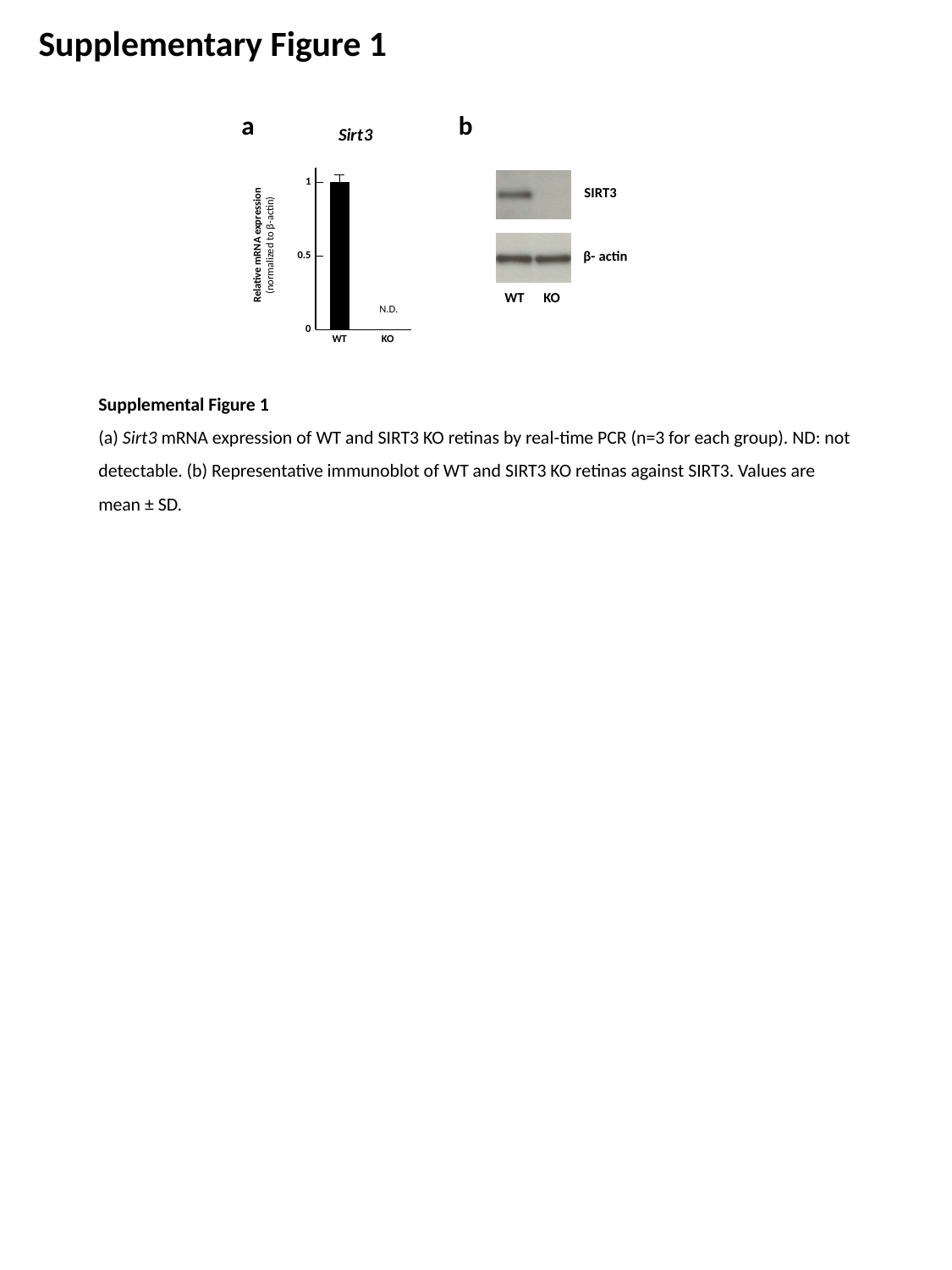

Supplementary Figure 1
b
SIRT3
β- actin
WT KO
a
### Chart: Sirt3
| Category | sirt3 |
|---|---|
| WT | 1.0 |
| KO | 0.00015518374717832337 |Relative mRNA expression
(normalized to β-actin)
N.D.
Supplemental Figure 1
(a) Sirt3 mRNA expression of WT and SIRT3 KO retinas by real-time PCR (n=3 for each group). ND: not detectable. (b) Representative immunoblot of WT and SIRT3 KO retinas against SIRT3. Values are mean ± SD.

## Slide 2
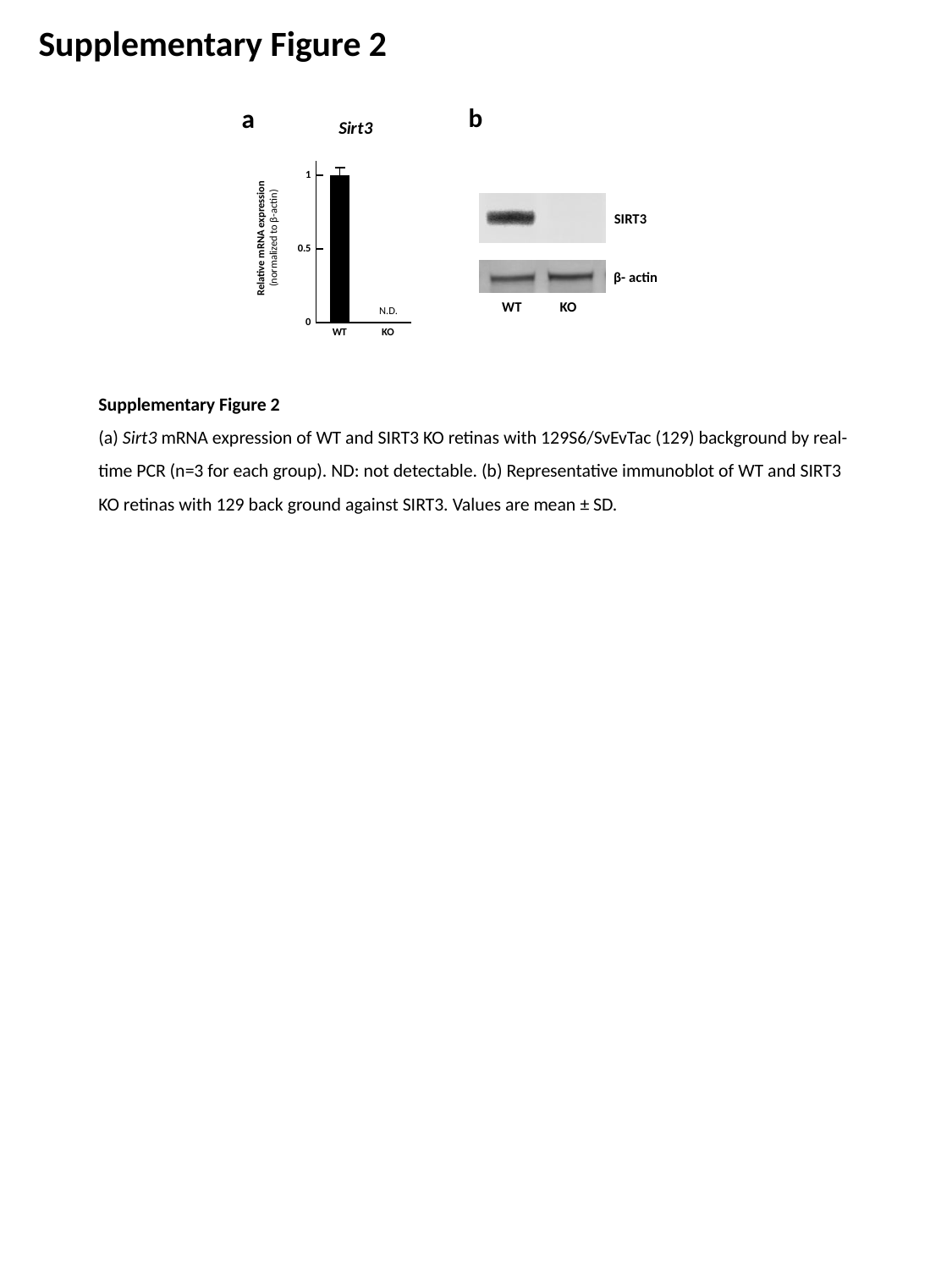

Supplementary Figure 2
b
SIRT3
β- actin
WT KO
a
### Chart: Sirt3
| Category | sirt3 |
|---|---|
| WT | 1.0 |
| KO | 0.00015518374717832337 |N.D.
Relative mRNA expression
(normalized to β-actin)
Supplementary Figure 2
(a) Sirt3 mRNA expression of WT and SIRT3 KO retinas with 129S6/SvEvTac (129) background by real-time PCR (n=3 for each group). ND: not detectable. (b) Representative immunoblot of WT and SIRT3 KO retinas with 129 back ground against SIRT3. Values are mean ± SD.

## Slide 3
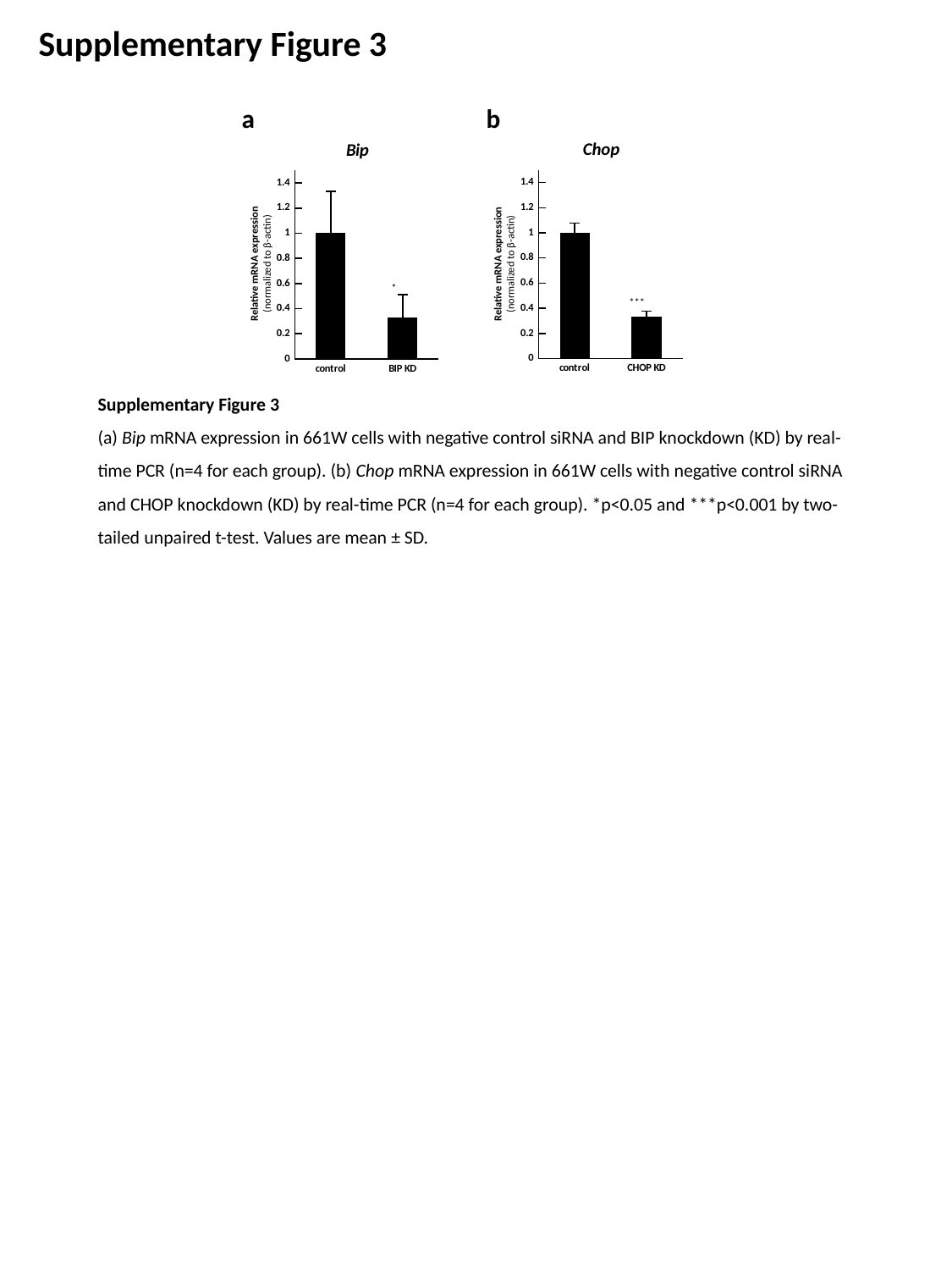

Supplementary Figure 3
a
### Chart: Bip
| Category | Nampt/β-actin mRNA |
|---|---|
| control | 1.0 |
| BIP KD | 0.32893312141036396 |*
Relative mRNA expression
(normalized to β-actin)
b
### Chart: Chop
| Category | Nampt/β-actin mRNA |
|---|---|
| control | 1.0 |
| CHOP KD | 0.3272595963624406 |***
Relative mRNA expression
(normalized to β-actin)
Supplementary Figure 3
(a) Bip mRNA expression in 661W cells with negative control siRNA and BIP knockdown (KD) by real-time PCR (n=4 for each group). (b) Chop mRNA expression in 661W cells with negative control siRNA and CHOP knockdown (KD) by real-time PCR (n=4 for each group). *p<0.05 and ***p<0.001 by two-tailed unpaired t-test. Values are mean ± SD.
